# Motor Function Deficits Following Chronic Prenatal Ethanol Exposure are Linked to Impairments in Insulin/IGF, Notch and Wnt Signaling in the Cerebellum

**Published:** 2013-01-01

**Authors:** Ming Tong, Jason Ziplow, William Cy Chen, Quynh-Giao Nguyen, Charles Kim, Suzanne M de la Monte

**Affiliations:** 1 Departments of Pathology (Neuropathology), Neurology, Neurosurgery and Medicine, USA; 2 Liver Research Center, USA; 3 Rhode Island Hospital, USA; 4 Brown University, USA; 5 Warren Alpert Medical School, Providence, RI, USA

**Keywords:** Fetal alcohol spectrum disorder, Insulin, IGF, Wnt, Notch, Cerebellum, Prenatal ethanol exposure, Signal transduction, Multiplex ELISA

## Abstract

**Background:**

Fetal alcohol spectrum disorder (FASD) is associated with deficits in cerebellar function that can persist through adolescence. Previous studies demonstrated striking inhibition of insulin and insulin-like growth factor (IGF) signaling in ethanol-exposed cerebella.

**Objectives:**

We sought to determine if FASD-induced impairments in motor function were associated with deficits in insulin/IGF signaling in juvenile cerebella. Given the growing evidence that insulin/IGF pathways cross-talk with Notch and Wnt to promote brain development and maturation; we also examined the integrity of canonical Wnt and Notch signaling networks in the brain following chronic prenatal ethanol exposure.

**Methods:**

Pregnant Long Evans rats were fed isocaloric liquid diets containing 0% or 24% ethanol by caloric content from gestation day 6 through delivery. Pups were subjected to rotarod testing on postnatal days (P) 15–16 and sacrificed on P30. Cerebella were used for molecular and biochemical analysis of insulin/IGF-1, canonical Wnt, and Notch signaling mechanisms.

**Results:**

Prenatal ethanol exposures impaired rotarod performance, inhibited signaling through insulin and IGF-1 receptors, IRS-1, and Akt, increased activation of GSK-3β, and broadly suppressed genes mediating the canonical Wnt and Notch networks.

**Conclusions:**

Abnormalities in cerebellar function following chronic prenatal ethanol exposure are associated with inhibition of insulin/IGF, canonical Wnt, and Notch networks that cross-talk via GSK-3β. Effective therapeutic measures for FASD may require multi-pronged support of interrelated signaling networks that regulate brain development.

## Introduction

Alcohol misuse during pregnancy causes neurodevelopmental defects, including microcephaly, cerebellar hypoplasia, motor deficits, and neuro-cognitive impairments ranging from attention deficit hyperactivity disorder to mental retardation. This constellation of abnormalities, together with a number of stereotypical craniofacial defects is termed, ‘fetal alcohol spectrum disorders’ (FASD) [1,2]. A major adverse effect of ethanol on the immature central nervous system (CNS) is to profoundly inhibit insulin and insulin-like growth factor (IGF) signaling [3].

Insulin and IGF regulate neuronal survival and differentiation, myelin formation and maintenance, neuronal migration, plasticity, metabolism, and neurotransmitter supply and responsiveness [4–12]. Ethanol inhibits insulin and IGF-1 receptor tyrosine phosphorylation, receptor tyrosine kinase activation, and insulin receptor substrate (IRS) signaling [13–15]. Importantly, IRS transmits growth, survival, and metabolic signals downstream through phosphotidyl-inositol-3-kinase (PI3K) and Akt, and PI3K/Akt inhibits glycogen synthase kinase 3β (GSK-3β) [3,14–19], which at high levels promotes oxidative stress and apoptosis [12]. Therefore, ethanol’s inhibition of insulin/IGF-1 during development could account for many of the structural and functional phenotypic features of FASD. On the other hand, it is unlikely that this concept reflects the full scope of the problem because many CNS developmental functions are regulated by other signaling networks, including Notch and Wnt, which cross-talk with insulin and IGF pathways [20,21]. Therefore, the adverse effects of prenatal ethanol exposure on CNS development, gene expression, and function could be due to broader impairments of signaling pathways that interconnect with insulin/IGF networks.

Wnt signaling is very complex due to the numerous ligands, receptors, and transcription factors that mediate its physiological functions. The 3 major Wnt signaling pathways are: canonical, Wnt/Calcium, and planar cell polarity [22–24]. In the canonical pathway, Wnt ligands, which comprise a large family of cysteine-rich secreted glycoproteins, bind to Frizzled receptors, inhibiting β-catenin degradation. Accumulation of β-catenin leads to its translocation to the nucleus where it interacts with T cell factor (TCF) transcription factors to regulate gene expression.

The Wnt/Calcium pathway, which is regulated by G coupled phospholipases and proteins, increases cytoplasmic free calcium, and activates protein kinase C, calcium calmodulin mediated kinase II (CK2), and calcineurin (phosphatase) [23,25]. The Wnt planar cell polarity pathway is activated by the RAS homologue gene family member A (RhoA) and RAC1, leading to stimulation of stress kinases such as Jun N-terminal kinase (JNK) and RHO-activated coil-containing protein kinase 1 (ROCK), with attendant remodeling of the cytoskeleton [26–30]. In the CNS, Wnt signaling modulates neuronal proliferation, migration, adhesion, differentiation, and axon outgrowth [31–34]. A potential role for ethanol-mediated impairment of Wnt in FASD was suggested by the recent findings that: 1) both canonical Wnt and insulin/IGF signaling regulate GSK-3β [35,36] and 2) Wnt cross-talks with insulin/IGF networks [20,37–39] through GSK-3β [40]. Mechanistically, ethanol’s activation of GSK-3β could impair Wnt signaling due to increased phosphorylation and attendant degradation of β-catenin [21,24,41–43].

Notch signaling modulates neurogenesis, differentiation, and cell fate during CNS development [44–46]. Notch receptors are located at the cell surface, and Notch signaling is activated by ligand, e.g. Delta, Serrate, Jagged, binding [47]. Subsequent proteolytic cleavage releases Notch’s intracellular domain, which translocates to the nucleus where it binds to transcriptional regulators [47,48] that increase expression of target genes including, Hairy-Enhancer of Split (HES) and HES-related proteins [49,50]. However, Notch signaling is regulated by aspartyl-(asparaginyl)-β-hydroxylase (AAH) [51–53], as Notch (receptor) and Jagged (ligand) have the consensus sequence for AAH hydroxylation [54,55]. AAH’s catalytic activity promotes Notch’s translocation to the nucleus, cell adhesion, and cell motility [56]. AAH is stimulated by insulin/IGF [57,58], and chronic prenatal exposure to ethanol inhibits AAH’s expression and catalytic activity in the developing brain [59].

**Figure 1:**
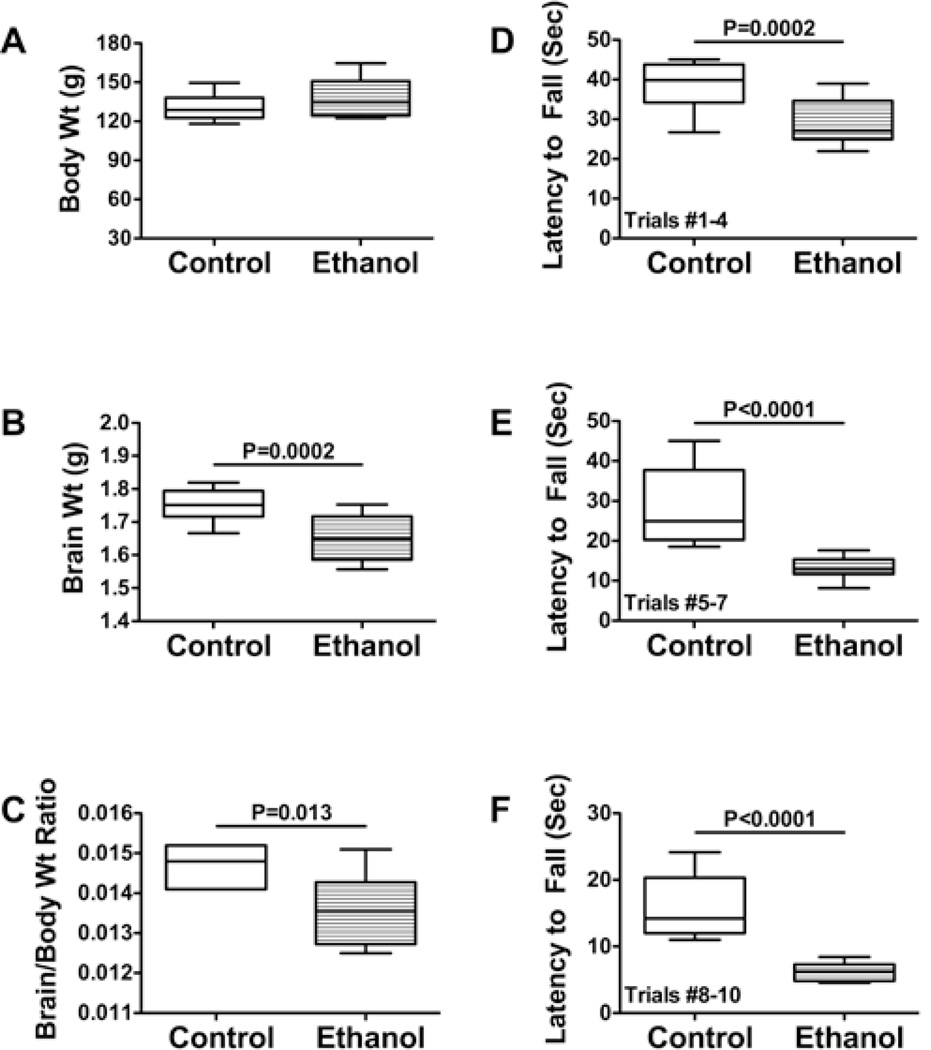
Effects of prenatal ethanol exposure on body growth, motor performance, and brain weight. Pregnant Long Evans rats were chronically fed with isocaloric liquid diets containing 0% or 24% ethanol. (A) Mean body weights, (B) brain weights, and (C) calculated brain weight/body weight ratios were determined on P30. The Student *t*-test was used to analyze data in Panels A–C. On P16, the rats were subjected to Rotarod testing with 10 incremental speed trials. Data from Trials (D) 1–4, (E) 5–7, and (F) 8–10 were culled and analyzed using the Mann-Whitney test. Significant inter-group differences are shown above the graphs.

The above discussion highlights critical roles for insulin/IGF, Wnt, and Notch signaling networks in brain development, and provides evidence that chronic prenatal ethanol exposures inhibit insulin/IGF and Notch signaling in immature brains. However, little is known about the effects of ethanol on Wnt signaling, and whether long-term impairments in CNS function caused by prenatal ethanol exposure are due to persistent inhibition of all 3 inter-connecting pathways. Our proposed model for how ethanol disrupts signaling and leads to structural, functional, and molecular abnormalities in the cerebellum is diagramed in figure 1. The present study addresses the overarching hypothesis by examining the integrity of insulin/IGF-1, Wnt, and Notch signaling in relation to the adverse effects of chronic prenatal ethanol exposure on cerebellar function in juvenile rats.

## Materials and Methods

### Materials

Qiazol reagent, EZ1 RNA universal tissue kit, QuantiTect SYBR Green polymerase chain reaction (PCR) master mix, Wnt array RT^2^-PCR Profiler and the BIO Robot Z1 were from Qiagen Inc. (Valencia, CA). Monoclonal antibodies to Notch-1, Jagged-1, β-Actin, HES-1, and β-catenin HES-1, and β-catenin were purchased from Abcam Inc. (Cambridge, MA) or Chemicon International (Tecumsula, CA). The A85G6 and A85E6 AAH monoclonal antibodies to AAH and Humbug, respectively, were generated to human recombinant protein [59] and purified over Protein G columns (Healthcare, Piscataway, NJ). Histofix was purchased from Histochoice (Amresco, Solon, OH). The AMV first strand cDNA synthesis kit was obtained from Roche Diagnostics Corporation (Indianapolis, IN). Enzyme-linked immunosorbent assay (ELISA) 96-well plates and the ELISA plate washer were purchased from Nunc (Rochester, NY). Horseradish peroxidase (HRP)-conjugated secondary antibody, Amplex Red soluble fluorophore, and the Akt Pathway Total and Phospho 7-Plex panels were purchased from Invitrogen (Carlsbad, CA). HRP-labeled polymer conjugated secondary antibody used for immunohistochemistry was purchased from Dako Corp (Carpentaria, CA). The SpectraMax M5 microplate reader was purchased from Molecular Devices Corp. (Sunnyvale, CA). Bicinchoninic acid (BCA) reagents were from Pierce Chemical Corp. (Rockford, IL). All other fine chemicals were purchased from CalBiochem (Carlsbad, CA), Pierce (Rockford, IL), or Sigma (St. Louis, MO).

### In utero ethanol exposure model

Twenty-four pregnant Long Evans rats were pair-fed with isocaloric liquid diets (BioServ, Frenchtown, NJ) in which ethanol comprised 0% (N=12) or 24% (N=12) of the caloric content. The diets were initiated on gestation day (GD) 6, and continued until parturition [60,61]. GD 0 was the day that sperm was detected in vaginal smears. The 24% ethanol containing diet produced early morning blood alcohol concentrations of 31.3 ± 8.3 µM. Ethanol feeding was begun on GD6 because earlier exposures lead to excessive fetal loss due to impaired placentation [62]. The dams were monitored to ensure equivalent caloric intake and body weight maintenance.

Once the pups were born, the litters were culled to 8 pups per dam, and maternal diets were switched to standard chow. Pups were housed with the dams until they were weaned. The pups were monitored daily and weighed weekly. The offspring were subjected to rotarod testing of motor function on postnatal days (P) 15-P16, and sacrificed on P30. Freshly harvested cerebella were snap frozen in a dry ice-methanol bath and stored at –80°C for RNA and protein studies. The cerebellum was studied because it is a major target of ethanol-induced neurotoxicity [1–3]. The Lifespan-Rhode Island Hospital IACUC committee approved these procedures and the use of rats in experiments.

### Rotarod testing

We used rotarod tests to assess effects of chronic pre-natal ethanol exposure on motor function [52,63]. On P15, rats were trained to remain balanced on the rotating Rotamex-5 apparatus (Columbus Instruments) at 1–5 rpm. All tested rats succeeded in the training. On P16, rats (N=12 per group) were administered 10 trials at incremental speeds up to 10 rpm, with 10 minutes rest between trials. The latency to fall was automatically detected and recorded with photocells placed over the rod. However, trials were stopped after 30 seconds to avoid exercise fatigue. Data from trials 1–3 (2–5 rpm), 4–7 (5–7 rpm), and 8–10 (8–10 rpm) were culled and analyzed using the Mann-Whitney test.

### Quantitative Reverse Transcriptase Polymerase Chain Reaction (qRT-PCR) analysis

We used qRT-PCR analysis to measure mRNA expression. Tissues were homogenized in Qiazol reagent, and total RNA was isolated using the RNAeasy Mini kit. RNA was reverse transcribed using the AMV First Strand cDNA synthesis kit and random oligodeoxynucleotide primers. The resulting cDNAs were used as templates in probe-based qPCR amplification reactions with gene-specific primer pairs. Primer-probe pairs were designed using ProbeFinder Version 2.45 software (Roche Applied Science, Indianapolis, IN), and mRNA target specificity was verified using NCBI-BLAST (Basic Local Alignment Search Tool). The amplified signals were detected and analyzed using the LightCycler 480 Real-Time PCR System and software (Roche Diagnostics, Indianapolis, IN). Expression levels for genes of interest were normalized to β-actin, which was measured simultaneously in the duplex qPCR reactions. Inter-group statistical comparisons were made using the calculated mRNA/β-actin ratios. In separate reactions, we determined that the mean levels of β-actin mRNA did not differ significantly for control and ethanol-exposed rats. The inclusion of primer-probe pairs to simultaneously measure β-actin enabled us to control for well-to-well differences caused by technical errors in pipetting, and also express levels of gene expression as relative abundance rather than fold differences.

### Duplex Enzyme Linked Immunosorbent Assay (ELISA)

Cerebellar tissue was homogenized in Nonidet-40 (NP-40) lysis buffer supplemented with protease (1 mM PMSF, 0.1 mM TPCK, 1 mg/ml aprotinin, 1 mg/ml pepstatin A, 0.5 mg/ml leupeptin, 1 mM NaF, 1 mM Na_4_P_2_O_7_) and phosphatase (2 mM Na_3_VO_4_) inhibitors using a Retsch Tissue Lyser (Newtown, PA). Samples were centrifuged at 15,000 g for 10 minutes, and the supernatants were used in assays of immunoreactivity. Protein concentrations were measured with the BCA assay. For direct binding ELISAs, 100ng of protein in 50 µl of bicarbonate buffer were adsorbed to the bottoms of MaxiSorp 96-well ELISA plates by overnight incubation at 4°C. After rinsing in Tris buffered saline (TBS; 50 mM Tris-HCl, pH 7.5, 150 mM NaCl), the wells were blocked for 3 hours with 250 µl/well of 2% bovine serum albumin (BSA) in TBS. The samples were then incubated with primary antibody (0.1–0.5 µg/ml) for 1 hour at 37°C. Immunoreactivity was detected with HRP-conjugated secondary antibody (1:10000) and Amplex UltraRed soluble fluorophore. Amplex Red fluorescence was measured (Ex 530/Em 590) in a SpectraMax M5 microplate reader (fluorescence light units; FLU). Subsequently, the samples were incubated with biotin-conjugated antibodies to large ribosomal protein (RPLPO), and immunoreactivity was detected with streptavidin-conjugated alkaline phosphatase (1:1000) and the 4-Methylumbelliferyl phosphate (4-MUP) fluorophore. Fluorescence (Ex360/Em450) intensity was measured in a SpectraMax M5. Non-specific binding was assessed with parallel negative control incubations in which the primary or secondary antibody was omitted. The mean ratios of specific protein/RPLPO fluorescence were used for statistical comparisons.

### Multiplex ELISA

We used bead-based multiplex ELISAs to examine the integrity of insulin and IGF-1 signaling networks by measuring immunoreactivity to the insulin receptor (IR), IGF-1 receptor (IGF-1R), IRS-1, Akt, GSK-3β, ^pYpY1162/1163^-IR, ^pYpY1135/1136^-IGF-1R, ^pS312^-IRS-1, ^pS473^-Akt, and ^pS9^-GSK3β according to the manufacturer’s protocol. Samples containing 200 µg of protein were incubated with the beads, and captured antigens were detected with biotinylated secondary antibody and phycoerythrin-conjugated Streptavidin. Plates were read in a Bio-Plex 200 system (Bio-Rad, Hercules, CA). Data are expressed as fluorescence light units corrected for protein concentration.

### Statistical analyses

To ensure inclusion of subjects with independent exposures to the control or ethanol-containing liquid diets, data from pupsin different litters (N=12 each) were analyzed in each group. Data corresponding to gene expression or immunoreactivity are depicted in boxplots representing the medians (horizontal bars), 95% confidence intervals (box limits), and range (whiskers) for each group. Planned inter-group comparisons were made using Student *t* tests. Multivariate analysis of variance (MANOVA) tests were used to identify the significant main effects of ethanol on: 1) upstream mediators of insulin and IGF signaling (trophic factor, receptor and IRS expression and phosphorylation); 2) downstream mediators of insulin/IGF signaling through Akt pathways; 3) Wnt genes and 4) Notch-related signaling genes. This approach was used to consolidate the large number of dependent variable responses in relation to ethanol exposure and correct for repeated measures. Statistical analyses were performed using the GraphPad Prism 5 (San Diego, CA) or NCSS8 (Kaysville, UT) software and significant P-values (<0.05) are indicated within graph panels or tables.

## Results

### Effects of chronic prenatal ethanol exposure on juvenile cerebellar motor function

In control and ethanol-exposed pups, body weights increased continuously, and at the end of the experiment (P30), the mean body weights were similar for the two groups (Figure 1A). Therefore, chronic prenatal ethanol exposure had no significant effect on postnatal body growth through early adolescence. In contrast, the mean brain weights (Figure 1B) and the calculated mean brain weight/body weight ratios (Figure 1C) were significantly reduced in the ethanol-exposed rats. Rotarod tests of cerebellar function demonstrated significantly shorter mean latencies to fall over all trial blocks, but the largest inter-group difference was observed with the most challenging trials (Figures1D–1F).

### Chronic prenatal ethanol exposure causes impairments in insulin/IGF signaling in the brain

**Figure 2:**
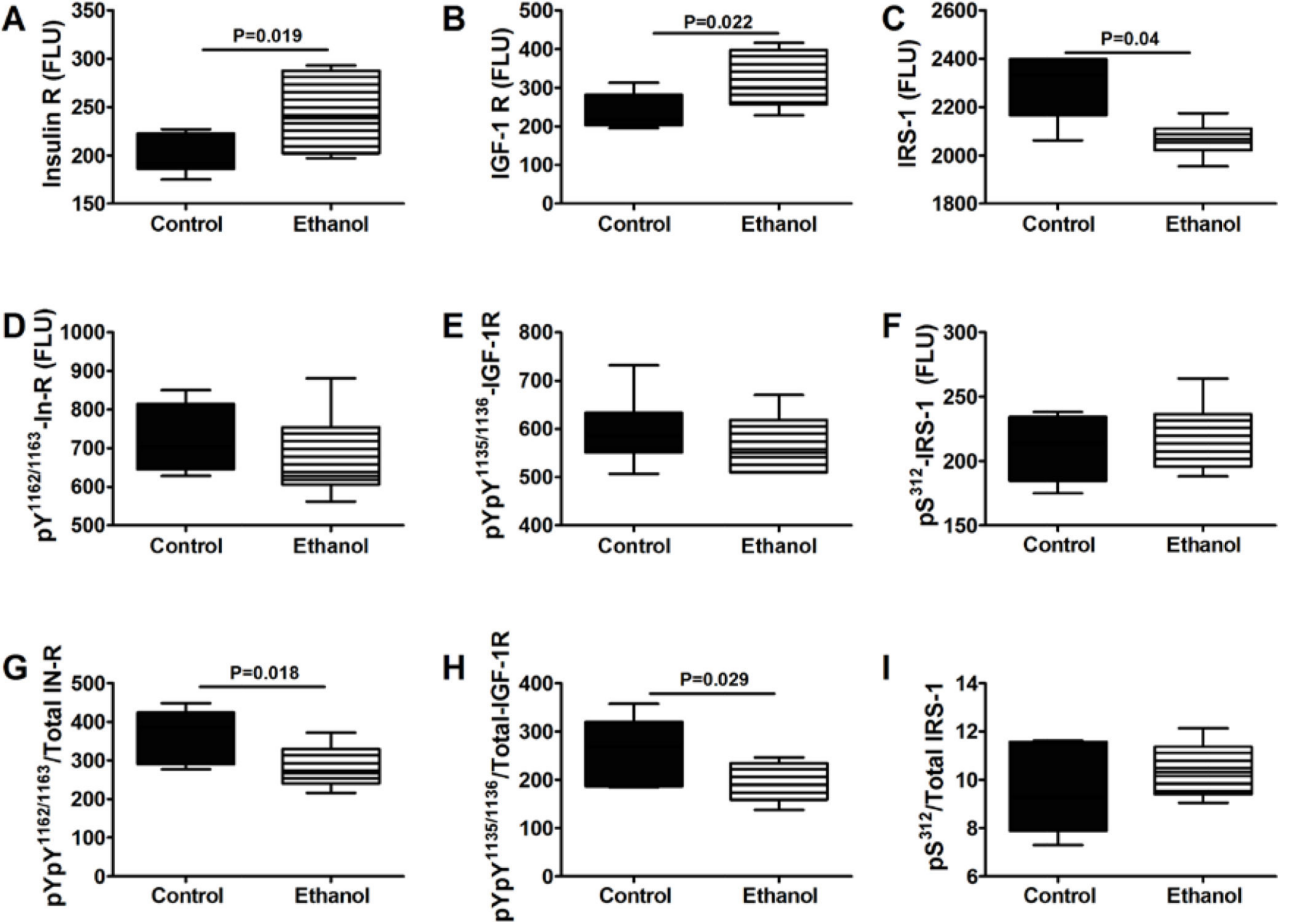
Cerebellar insulin and IGF-1 resistance following chronic prenatal ethanol exposure. P30 cerebella were used to measure Immunoreactivity to the (A) insulin receptor (IR), (B) IGF-1R, (C) IRS-1, (D) ^pYpY1162/1163^-IR, (E) ^pYpY1135/1136^-IGF-1R, (F) ^pS312^-IRS-1 by multiplex ELISA. Phospho-/total protein ratios for (G) IR, (H) IGF-1R, and (I) IRS-1 were calculated. Inter-group comparisons were made using Student *t*-tests. Significant differences are indicated within the panels. N=12/group with each sample contributed from a different litter.

We used multiplex ELISAs to interrogate ethanol’s effects on the integrity of brain insulin/IGF signaling. To examine upstream pathway components, we measured total and phosphorylated levels of insulin receptor, IGF-1 receptor, and IRS-1, and calculated the relative levels of phosphorylation from the ratios of phospho-/total protein (Figure 2). Chronic prenatal ethanol exposure resulted in higher mean levels of insulin and IGF-1 receptor and lower levels of IRS-1 protein (Figures 2A–2C). Although there were no significant inter-group differences in the levels of tyrosine phosphorylated (activated) insulin and IGF-1 receptors (Figures 2D and 2E), the relative levels of tyrosine phosphorylated insulin and IGF-1 receptors were significantly reduced by prenatal ethanol exposure (Figures 2G and 2H). Multivariate ANOVA (MANOVA) testing demonstrated a significant main effects of ethanol on insulin receptor (F=5.53; P=0.04), ^pYpY1162/1163^-IR/total IR (F=5.91; P=0.035), and ^pYpY1135/1136^-IGF-1R/total IGF-1R (F=4.92; P=0.05), and a main effect trend of ethanol on IGF-1 receptor (F=2.96; P=0.10) expression. Therefore, the chronic prenatal ethanol exposures caused insulin and IGF-1 resistance (increased receptor expression vis-à-vis reduced receptor tyrosine phosphorylation) in the brain. Although the mean levels of ^pS312-^IRS-1 and ^pS312-^IRS-1/total IRS-1 were similar in control and ethanol-exposed cerebella (Figures 2F and 2I), the reduced levels of IRS-1 protein vis-a-vis decreased activation of insulin and IGF-1 receptor tyrosine kinases could have further impaired insulin and IGF-1 downstream signaling.

### Effects of prenatal ethanol exposure on downstream signaling through Akt and GSK-3β

**Figure 3:**
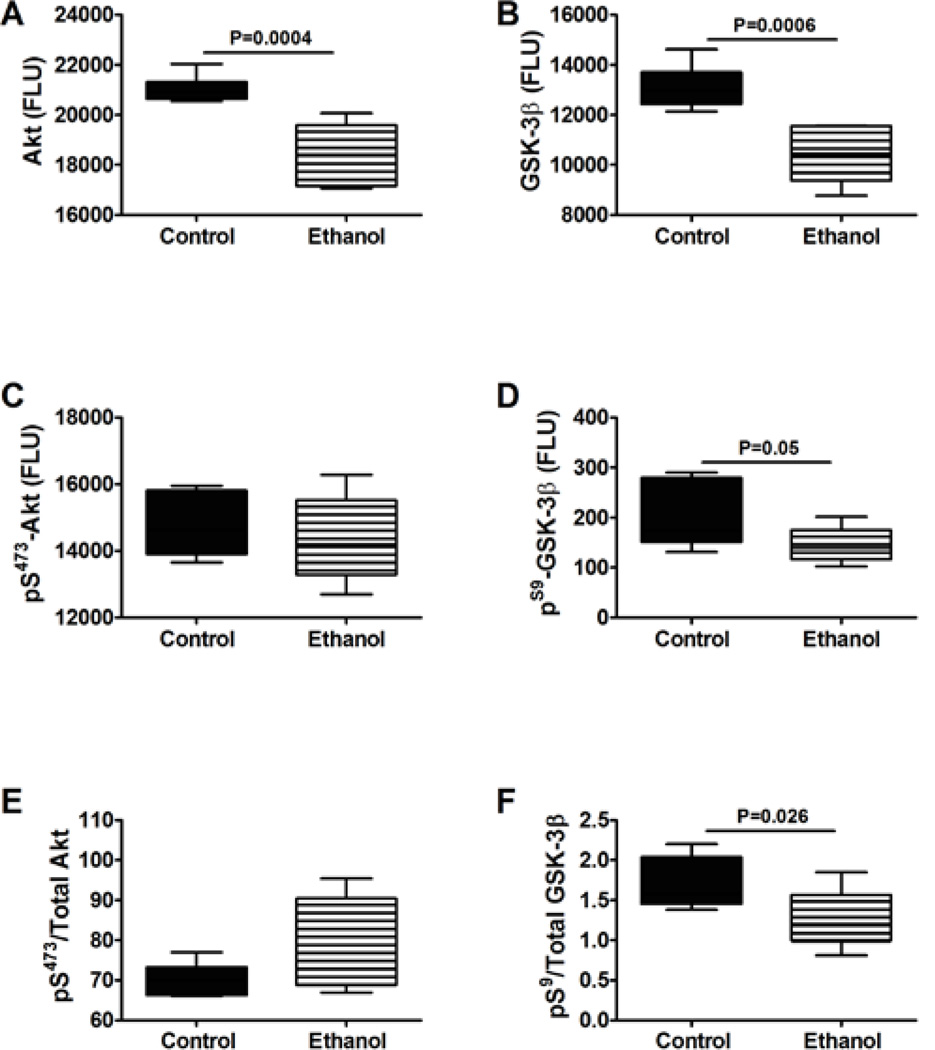
Long-term effects of chronic prenatal ethanol exposure on signaling through Akt and GSK-3β. P30 cerebellar protein homogenates were used to measure (A) Akt, (B) GSK-3β, (C) ^pS473^-Akt, and (D) ^pS9^-GSK3β immunoreactivity by multiplex ELISA. (E,F) Phospho-/total protein ratios were calculated. Inter-group comparisons were made using Student *t*-tests. Significant differences are indicated within the panels. N=12/group with each sample contributed from a different litter.

Insulin, IGF-1 and IRS-1 signals downstream to activate Akt and inhibit GSK-3β through phosphorylation of specific Ser residues on these proteins. In addition, signaling through Akt and GSK-3β can be regulated by the levels of protein expression. Multiplex ELISAs demonstrated that chronic prenatal ethanol exposures significantly reduced cerebellar levels of total Akt, but not ^pS473-^Akt or ^pS473^/total Akt (Figures 3A,3C and 3E). Ethanol also significantly reduced the mean levels of GSK-3β, ^pSer-9^-GSK-3β, and the ^pSer-9^-/total GSK-3β ratio (Figures 3B,3D and 3F). MANOVA demonstrated significant main effects of ethanol effects on Akt (F=20.85; P=0.001), GSK-3β (F=12.11; P=0.006), and ^pSer-9^-GSK-3β/total GSK-3β (F=4.99; P=0.05). Since GSK-3β is inactivated by Ser-9 phosphorylation, the net effect of prenatal ethanol exposure was to increase GSK-3βactivity in juvenile cerebella.

### Prenatal ethanol exposure impairs Wnt signaling in juvenile brains

**Figure 4:**
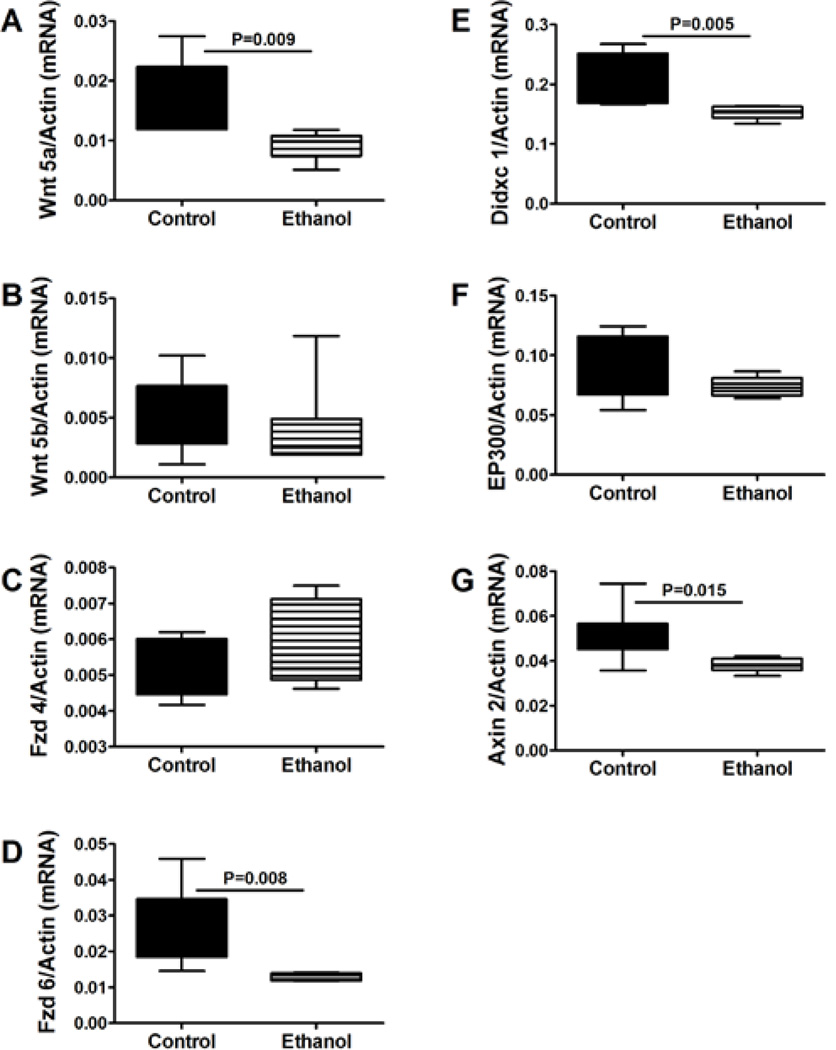
Effects of chronic prenatal ethanol exposure on Wnt signaling in juvenile cerebella. Probe-based qPCR amplification reactions were used to measure (A) Wnt 5a, (B) Wnt 5b, (C) Fzd 4, (D) Fzd 6, (E) Didxc 1A, (F) EP300, and (G) Axin 2. Results were normalized to β-actin, which was measured simultaneously in the duplex qPCR reactions. Inter-group comparisons were made with calculated mRNA/β-actin ratios. N=12/group with each sample contributed from a different litter.

Since insulin/IGF-1 signaling cross-talks with Wnt [20,37–39], we hypothesized that the impaired insulin/IGF-1 signaling in cerebella of chronic prenatal ethanol exposed rats would be associated with down-regulation of Wnt pathway genes (Figure 1). We first performed exploratory qPCR studies in which the expression levels of 84 Wnt pathway and target genes (Supplementary tables 1 and 2) were compared between pooled samples of P20 control and ethanol-exposed rats (N=3 per group with each sample obtained from different litters) using the same model described in the Materials and Methods section (Supplementary Methods). The results suggested that chronic prenatal ethanol exposure inhibits Wnt signaling at all levels of the canonical pathway. From the list of genes in which inter-group differences were 2-fold or greater, or expression levels were relatively high compared with other isoforms of the corresponding ligands or receptors, we focused our further analyses by measuring expression of: Wnt 5a, Wnt 5b, Frizzled (Fzd) 4, Fzd 6, Didxc, EP300, and Axin2 (Figure 4). Chronic prenatal ethanol exposure significantly reduced expression of Wnt5a, Fzd 6, Didxc, and Axin 2, and caused modest but not statistically significant reductions in Wnt 5b and Ep300 in cerebellar tissue. In contrast, Fzd 4 mRNA levels were similar in the control and ethanol-exposed groups. MANOVA testing demonstrated significant main effects of ethanol exposure on Fzd 6 (F=8.45; P=0.015), Didxc (F=9.86; P=0.01), and Axin 2 (F=6.47; P=0.029). Corresponding with the broad inhibition of Wnt pathway genes, ELISA studies demonstrated significantly reduced levels of β-catenin in ethanol-exposed relative to control cerebella (Table 1).

### Chronic prenatal ethanol exposure inhibits Notch pathway genes in juvenile cerebella

**Figure 5:**
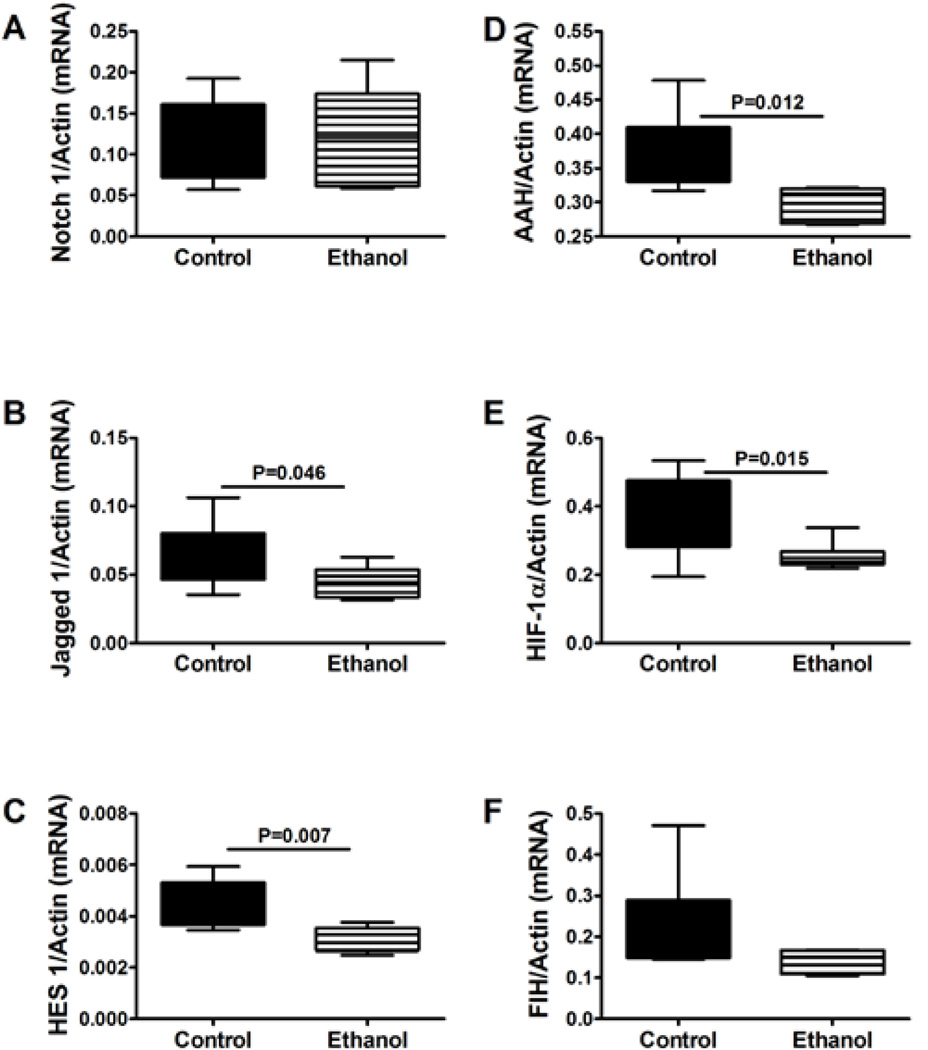
Effects of chronic prenatal ethanol exposure on Notch signaling in juvenilecerebella. Probe-based qPCR amplification reactions were used to measure (A) Notch 1, (B) Jagged 1, (C) HES 1, (D) AAH, (E) HIF-1α, and (F) FIH gene expression with results normalized to β-actin, which was measured simultaneously. Inter-group comparisons were made using the calculated mRNA/β-actin ratios. N=12/group with each sample contributed from a different litter.

Insulin and IGF-1 signaling regulate AAH and hypoxia-inducible factor-1a (HIF-1α) expression [51], and both AAH and HIF-1α cross-talk with Notch [51]. Activation of insulin/IGF-1, AAH, Notch, and HIF-1α promotes cell motility [51–53,58,64], whereas ethanol inhibition of insulin/IGF-1 signaling and AAH impairs neuronal migration [59,65]. We extended our investigations to determine the degree to which chronic prenatal ethanol exposure impairs Notch signaling in juvenile cerebella. Moreover, we examined expression of other genes and proteins that cross-talk within this network and regulate neuronal motility and/or adhesion, i.e. Humbug (AAH-related molecule that lacks the C-terminal catalytic region) [54], HIF-1α, and factor inducing HIF-1α (FIH) [51]. In these studies, we measured Notch 1, Jagged 1, and HES-1, AAH, HIF-1α, and FIH mRNAs in P30 cerebella. Probe-based qRT-PCR analysis demonstrated significantly lower levels of Jagged 1, HES-1, AAH, and HIF-1α expression in ethanol-exposed relative to control brains. In contrast, no significant inter-group differences were observed with respect to Notch 1 or FIH (Figure 5). MANOVA testing demonstrated significant main effects of ethanol on HIF-1α (F=6.37; P=0.03), AAH (F=19.26; P=0.001), and HES-1 (F=8.95; P=0.014), and main effect trends with respect to Jagged (F=3.47; P=0.09) and FIH (F=3.75; P=0.081). Furthermore, ELISAs demonstrated significantly reduced levels of Jagged 1, Humbug, and AAH, in ethanol-exposed versus control cerebella (Table 1).

## Discussion

### Chronic prenatal ethanol exposure impairs insulin/IGF-1 signaling in juvenile cerebella

This study demonstrates that motor impairments caused by chronic prenatal ethanol exposure, were associated with persistent insulin/IGF-1 resistance which is characterized by elevated levels of insulin/IGF-1 receptor expression vis-à-vis reduced levels of receptor tyrosine phosphorylation. The associated inhibition of IRS-1 expression could have further impaired insulin/IGF-1 signaling by restricting the substrate needed to transmit signals to downstream pathways. Although it is not readily apparent why brain insulin/IGF-1 resistance persists beyond the period of ethanol exposure, consequences would include ongoing cell loss due to impaired survival mechanisms, increased oxidative stress, and deficits in cholinergic function [61]. The reduced IRS-1 protein levels could have been mediated by insulin/IGF-1 resistance, since insulin and IGF-1 regulate IRS gene expression [66–68].

Insulin/IGF-1 resistance typically reduces Akt [15,18,19,69–71], and increases GSK-3β [19,59,65,72] activity. Paradoxically, in ethanol-exposed cerebella, ^pS473-^Akt levels were not reduced, indicating that the kinase activation mechanisms were intact. On the other hand, Akt protein expression was significantly reduced in ethanol-exposed cerebella; previous studies showed that Akt signaling can be modulated via changes in its protein levels [73,74]. Therefore, the combined effects of insulin/IGF-1 resistance and reduced Akt expression could account for the persistent structural and functional abnormalities observed in ethanol-exposed juvenile cerebella. Moreover, the deleterious effects of insulin/IGF-1 resistance on growth, metabolism, and cell survival were likely exacerbated by activation of GSK-3β (reduced Ser9 phosphorylation), and attendant increased oxidative stress and pro-death signaling in ethanol-exposed cerebella.

### Prenatal ethanol exposure impairs Wnt signaling in juvenile brains

Wnt signaling helps mediate brain morphogenesis, cell proliferation, migration, differentiation, and axonal outgrowth, and it cross-talks with insulin/IGF pathways. However, the available data about ethanol’s effects on Wnt signaling in the developing brain are limited. Previous studies showed that chronic prenatal followed by postnatal ethanol exposures lead to persistently high brain levels of Wnt and β-catenin protein, which would impair neuronal maturation [75,76]. Our study showed that the long-term effects of just chronic prenatal ethanol exposures include inhibition of genes at multiple levels of the canonical Wnt signaling pathway, i.e. Wnt ligands (Wnt 5a), receptors (Frizzled 6), and components of the destruction complex (Didxc and Axin 2), and decreased β-catenin immune reactivity. Since insulin/IGF-1 networks cross-talk with Wnt via GSK-3β [21,35,39,77], increased GSK-3β activity caused by insulin/IGF-1 resistance could have further impaired Wnt signaling in juvenile cerebella. Mechanistically, activated GSK-3β phosphorylates β-catenin, causing it to be targeted for degradation via the ubiquitin-proteasome pathway [24,43,77]. Correspondingly, β-catenin protein levels were significantly reduced in chronic ethanol-exposed brains. Therefore, the long-term adverse effects of chronic prenatal ethanol exposures on cerebellar development and function are likely mediated in part by inhibition of gene expression and disruption of insulin/IGF cross-talk with the canonical Wnt pathway.

### Effects of chronic prenatal ethanol exposure on Notch pathway genes

Notch signaling mediates neuronal migration and plasticity [44–46,78]. Therefore, inhibition of Notch could result in significant structural and functional abnormalities in the developing brain. Notch signaling is activated by AAH [53,56,58,64,79], as both Notch and Jagged proteins contain the consensus sequence for AAH’s catalytic activity [54,56]. AAH promotes cell motility, and its expression and catalytic activity are regulated by insulin/IGF-1 [59]. Correspondingly, ethanol inhibition of insulin/IGF-1 signaling decreases AAH expression and catalytic activity, and impairs cerebellar neuron migration [59], but the effects on Notch have not yet been determined. AAH and Notch are also regulated by HIF-1α which, in addition hypoxia, is regulated by insulin/IGF-1 [51]. Therefore, Notch, Jagged, AAH, and HIF-1α signaling networks are inter-related through insulin/IGF-1(Figure 1) [51,52].

The studies herein demonstrate that chronic prenatal ethanol exposure causes inhibition of Notch (reduced Jagged and HES-1 expression), HIF-1α, AAH and insulin/IGF-1 signaling in juvenile cerebella. Reduced AAH and HIF-1α were likely mediated by insulin/IGF-1 resistance, whereas reduced Jagged-1 expression was probably mediated by inhibition of AAH [51]. Jagged-1 is a ligand for Notch receptor, and impaired expression of Notch ligands inhibits Notch signaling [80]. Decreased HES-1 mRNA corresponds with ethanol’s inhibitory effects on Notch signaling, as HES-1 is a downstream target gene of Notch [49,50].

**Figure 6:**
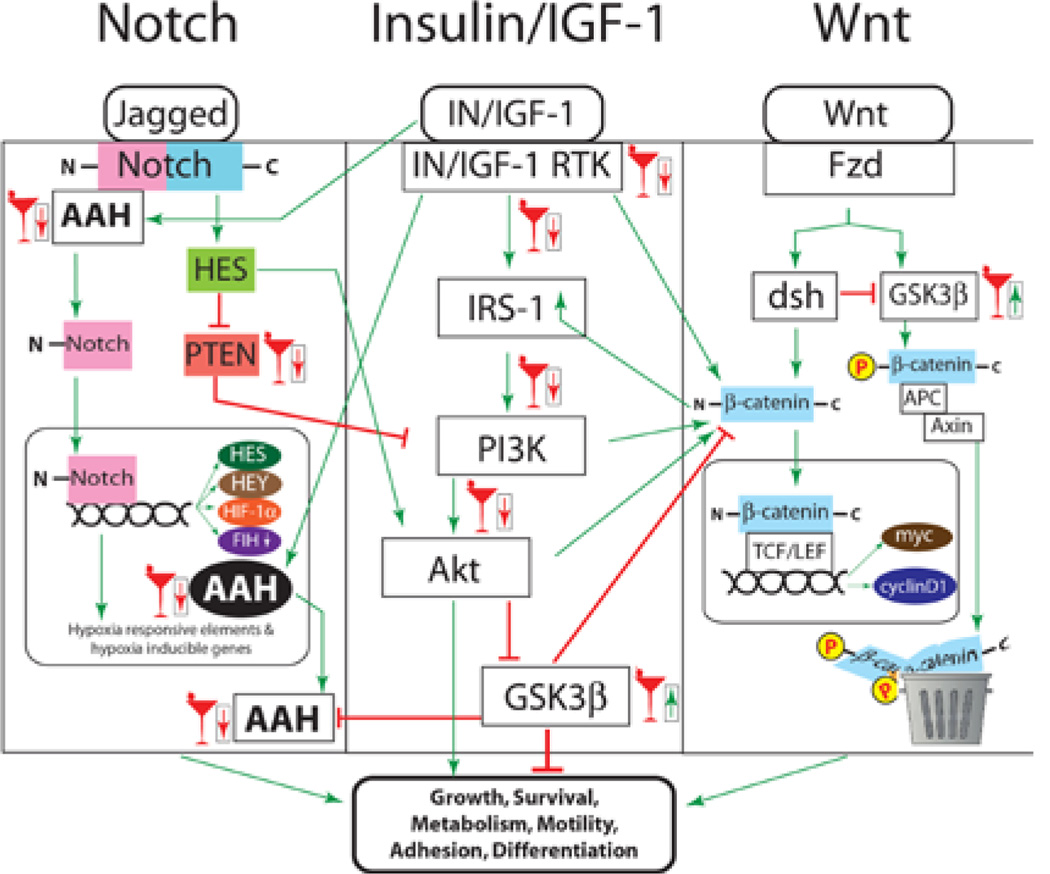
Proposed effects of ethanol on cross-talk signaling of insulin/IGF-1 with Notch and Wnt pathways in the developing brain. Ethanol inhibits insulin/IGF-1 signaling by impairing ligand-receptor binding, activation of receptor tyrosine kinases, tyrosine phosphorylation of IRS-1, and transmission of signals downstream through Akt pathways. In addition, ethanol activates phosphatases (PTP-1b and PTEN) that negatively regulate signaling through insulin/IGF-1 receptors and PI3K. Ethanol inhibits insulin/IGF-1 regulated genes that mediate Notch signaling, e.g. AAH. The net effect is inhibition of Notch target genes such as HES and HEY, and reduced cross-talk with the insulin/IGF-1 pathways via inhibition of PTEN and activation Akt. Ethanol disrupts Notch signaling at other levels in the cascade, including via ligand and receptor gene expression. Canonical Wnt signaling is regulated by GSK-3β, which phosphorylates β-catenin, targeting it for proteolytic destruction. Ethanol may impair Wnt signaling by inhibiting insulin/IGF-1 suppression of GSK-3β or by directly activating GSK-3β. The resulting high levels of GSK-3β activity could dampen positive cross-talk with Wnt-activated cellular functions.

These results demonstrate that the functional cerebellar abnormalities in prenatal ethanol-exposed juvenile rats are associated with inhibition of signaling through three major inter-related signaling pathways: insulin/IGF-1, canonical Wnt and Notch. This suggests that the pathogenic basis for the neurodevelopmental defects in FASD is complex and mediated by disruption of several major inter-connected signaling networks that cross-talk through insulin/IGF-1 and GSK-3β. Our proposed model of how these three pathways cross-talk and how ethanol disrupts their signaling is diagramed in figure 6. The limitations of the study design are: 1) we did not validate the results by treating the rats with inhibitors or activators of insulin, Wnt, or Notch signaling; 2) it is not possible from the results to determine whether the impairments in Wnt and Notch signaling are caused by insulin/IGF resistance, or vice versa; and 3) it is not possible to determine the degree to which specific impairments in insulin, Wnt, or Notch signaling mediate the phenotypic abnormalities in our model. The availability of specific inhibitors and/or activators of insulin, Wnt, and Notch signaling will enable these questions to be addressed mechanistically. Studies currently in progress are designed to determine the degree to which restoration of insulin/IGF signaling with insulin sensitizer agents abrogates impairments in Notch and Wnt signaling, together with the structural and functional abnormalities in the cerebellum. However, due to the complex nature of the cross-talk among several major signal transduction networks, we anticipate that multi-pronged therapeutic approaches will be required to prevent or reduce the severity of deficits caused by prenatal ethanol exposure.

## Figures and Tables

**Table 1:** Effects of Chronic Prenatal Ethanol Exposure on Notch and Wnt Pathway Protein Expression in Cerebellum.

Immunoreactivity	Control	Ethanol	*t*-Test
Notch 1	20002.8 ± 1182.0	19892.7 ± 1028.2	
Jagged 1	12360.8 ± 590.9	9897.8 ± 1031.3	0.035
HES-1	3883.5 ± 111.2	3917.7 ± 159.2	
Humbug	18863.4 ± 1007.0	13628.9 ± 1318.8	0.003
AAH	10340.2 ± 491.6	7581.0 ± 710.3	0.003
β-Catenin	4824.5 ± 138.1	3828.6 ± 227.2	<0.001

Immunoreactivity was measured in P30 cerebellar tissue by duplex ELISA with results normalized to large ribosomal protein measured in the same wells. Results correspond to mean net fluorescence light units (± S.E.M.) in 50 ng protein. Data were generated from 12 rats per group, with each sample contributed from a different litter. Inter-group comparisons were made with Student T-tests
